# Sociodemographic and Clinical Profile of Patients Referred to Dementia Outpatient Clinics in Tertiary Hospitals in Porto Alegre

**DOI:** 10.1002/alz70857_106160

**Published:** 2025-12-25

**Authors:** Augusto Colferai Marcon, Sarah Vitoria Bristot Carnevalli, Pedro Hall Ruschel, Liana Lisboa Fernandez, Alvaro de Oliveira Franco, Raphael Machado Castilhos

**Affiliations:** ^1^ Universidade Federal do Rio Grande do Sul, Porto Alegre, Rio Grande do Sul, Brazil; ^2^ Universidade Federal de Santa Catarina, Florianópolis, Santa Catarina, Brazil; ^3^ Universidade Federal de Ciências da Saúde de Porto Alegre (UFCSPA), Porto Alegre, Rio Grande do Sul, Brazil; ^4^ Hospital de Clinicas de Porto Alegre, Porto Alegre, Rio Grande do Sul, Brazil; ^5^ Hospital de Clínicas de Porto Alegre, Porto Alegre, Rio Grande do Sul, Brazil

## Abstract

**Background:**

Dementia is a syndrome characterized by progressive deterioration of cognitive functions and functionality, typically caused by neurodegenerative conditions. Dementia is typically associated with aging, responsible for disability and dependence, and is an even greater problem in low‐ and middle‐income countries, where 2/3 of the global dementia population resides Thus, the objective of this study is to evaluate the profile of patients referred from primary care to specialized dementia services in the city of Porto Alegre, in addition to assessing possible impacts of the COVID‐19 pandemic on the referral process.

**Method:**

Cross‐sectional study that analyzed the sociodemographic and clinical profile of patients treated as a first consultation in dementia outpatient clinic from 2019 and 2022, in two tertiary hospitals in the city of Porto Alegre: Hospital de Clínicas de Porto Alegre (HCPA) and Irmandade Santa Casa de Misericórdia de Porto Alegre (ISCMPA). Only first‐time consultations of patients from the public primary care network were included and the diagnosis was collected based on the patient's last consultation.

**Result:**

In total 251 patients from first visits were analyzed, 119 in 2019 and 132 in 2022. There was a significant increase in the median age and onset age of first symptoms: 70 years in 2019 and 73 years in 2022 (*p* < 0.001), 66 (IQR = 60‐72.5) in 2019 to 69 (IQR = 63‐75) in 2022 (*p* = 0.009), respectively. The median duration of symptoms doubled, being 1 year in 2019 and 2 years in 2022, albeit not significant. The median score of the Mini Mental State Examination (MMSE) was 18 in both years and there is a significant number of psychiatric diagnoses, 23.5% in 2019 and 16.7% in 2022.

**Conclusion:**

The data suggest a delay in the diagnosis and specialized care for patients with demential symptoms from public health, which was more expressive after the COVID‐19 pandemic. In addition, there is a high prevalence of psychiatric diagnosis, which demonstrates a possible misreferral from primary care.

